# Spontaneous Sublingual Hematoma in a COVID-19 Patient on Heparin Therapy: A Case Report and Review of Management Challenges

**DOI:** 10.1155/carm/3371235

**Published:** 2025-05-24

**Authors:** Mohsen Golkar, Anita Taheri, Milad Baseri, Parnian Nikraftar, Ramin Ansari, Ardeshir Khorsand

**Affiliations:** ^1^Department of Oral and Maxillofacial Surgery, Dentistry Faculty, Shahid Beheshti University of Medical Sciences, Tehran, Iran; ^2^Sina Trauma and Surgery Research Center, Tehran University of Medical Sciences, Tehran, Iran; ^3^Department of Radiology, Medicine Faculty, Shahid Beheshti University of Medical Sciences, Tehran, Iran

**Keywords:** anticoagulant therapy, COVID-19, heparin therapy, sublingual hematoma

## Abstract

COVID-19 is associated with a hypercoagulable state, often managed with anticoagulation therapy to prevent thrombotic events. However, anticoagulation can lead to rare but serious bleeding complications. We present the case of a 62-year-old male with severe COVID-19 admitted to the intensive care unit (ICU) with shortness of breath, cough, and oxygen desaturation. Also, he had diabetes, undergoing treatment with Neutral Protamine Hagedorn (NPH) insulin, and ischemic heart disease. On the eighth day of his admission, he developed a spontaneous sublingual hematoma while on unfractionated heparin therapy. The patient was managed conservatively with blood pressure control, cold compresses, adjustment of anticoagulation and close monitoring with laboratory anticoagulation tests, and careful observation. Despite the hematoma's initial enlargement, he exhibited no respiratory distress, and the hematoma gradually resolved without surgical intervention. This case highlights the need for vigilant monitoring, careful management of anticoagulation, and a multidisciplinary approach in balancing the benefits and risks of anticoagulation in COVID-19 patients.

## 1. Introduction

COVID-19, caused by the SARS-CoV-2 virus, emerged in late 2019 and quickly became a global pandemic. It spreads primarily through respiratory droplets and can cause a range of symptoms from mild respiratory issues to severe illness and death, particularly in the elderly and those with underlying conditions [1]. COVID-19 has been associated with significant alterations in the microcirculation, which can contribute to the pathogenesis of organ injury [2]. The infection often leads to a hypercoagulable state, characterized by widespread pulmonary microvascular thrombosis and, in some cases, extrapulmonary microvascular involvement [3]. Studies have shown that patients with severe COVID-19 exhibit increased vascular density and decreased flow velocity, leading to impaired tissue perfusion [4].

Given the hypercoagulable state induced by COVID-19, anticoagulant therapy has become a critical component of the treatment regimen to prevent thromboembolic events. The World Health Organization (WHO) recommends the use of anticoagulants such as heparin or low-molecular–weight heparin (LMWH) for thromboprophylaxis in hospitalized patients with COVID-19 [5]. This approach is based on the increased risk of thromboembolism due to cytokine storm and systemic inflammatory response syndrome (SIRS) that alter the coagulation profile [3].

Despite the benefits of anticoagulant therapy, it is not without risks. Hemorrhagic complications, including spontaneous hematomas, can occur in patients receiving anticoagulation [6]. These complications are particularly concerning in COVID-19 patients due to their already compromised health status [7].

Spontaneous hematomas, particularly in critical regions such as the neck and sublingual areas, pose a significant clinical challenge. These hematomas can lead to airway obstruction and other severe complications, necessitating prompt diagnosis and management. The occurrence of such hematomas underscores the need for careful monitoring of coagulation parameters and vigilant management of anticoagulant therapy in COVID-19 patients [8].

In this case report, we describe a 62-year-old male with COVID-19 who developed a spontaneous sublingual hematoma while receiving unfractionated heparin for anticoagulation. This case highlights the delicate balance required in managing anticoagulation to prevent thromboembolic events while minimizing the risk of hemorrhagic complications.

## 2. Case Report

**Figure 1:**
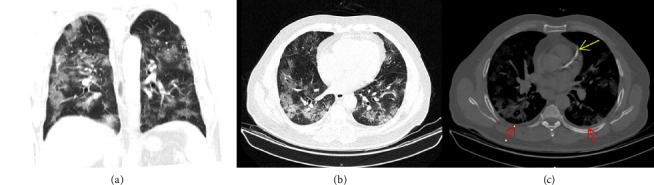
In without contrast, a chest CT scan taken on the day of admission that diffuse ground glass alveolar opacities in both lungs dominantly in the peripheral zones were considered in favor of viral pneumonia. (a) Coronal view. (b) Axial view. (c) Bilateral minimal pleural effusion is seen in this image (red arrows). Also, note the coronary artery stent (yellow arrow).

A 62-year-old male with a history of diabetes mellitus and ischemic heart disease was admitted to the intensive care unit (ICU) due to severe COVID-19 (Figure 1). His manifestations were cough, shortness of breath, and oxygen desaturation. Upon admission, he was started on unfractionated heparin therapy (7500 units) to manage the hypercoagulable state associated with COVID-19, as part of a routine empirical anticoagulation protocol established by the internal medicine department of the hospital for COVID-19 patients. Also, N-acetyl cysteine (NAC) 600 mg PO BD and Seroflo 2 puff BD were transcribed for alleviating the COVID-19 symptoms. Furthermore, pantoprazole 40 mg PO daily was considered for gastrointestinal prophylaxis. Besides, he was undergoing treatment with 26 units of Neutral Protamine Hagedorn (NPH) insulin subcutaneously for his diabetes. It is noteworthy that he was under treatment with acetylsalicylic acid (ASA) 80 mg PO daily and Aldactone 25 mg PO daily for his ischemic heart disease.

On the eighth day of his ICU stay, the patient experienced a mild episode of epistaxis, which resolved promptly. Later that day, he developed swelling in the floor of his mouth, raising suspicion of a sublingual hematoma. Examination confirmed a sublingual hematoma without active bleeding from the mouth or nose, and multiple clots were noted in the nasal passages without an active bleeding site. The patient exhibited stable vital signs, with a blood pressure of 116/69 mmHg, a heart rate of 87 bpm, and an oxygen saturation of 94% with supplemental oxygen and 70% without oxygen.

**Figure 2:**
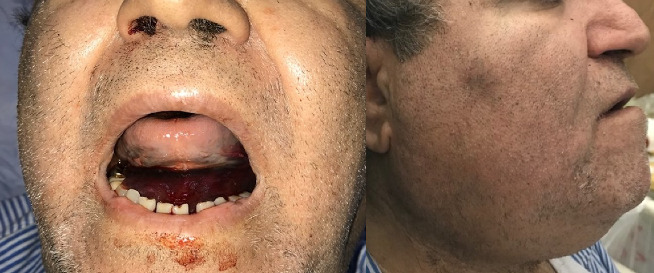
Clinical photograph of the floor of the mouth showing a prominent sublingual hematoma. The swelling is visible beneath the tongue, causing elevation and discoloration, consistent with hematoma formation in the sublingual space.

Within hours after the hematoma was discovered, it was observed to be enlarging (Figure 2). Despite this increase in size, the patient showed no signs of dyspnea (difficulty breathing) or respiratory distress. He reported mild dysphagia and odynophagia but had no drooling, and his airway remained open with the trachea in the midline. He was alert, oriented, and followed commands. Oral examination revealed no erythema, exudate, or aphthous ulcers, and facial nerve function was normal bilaterally. Eye examination showed a normal range of motion (ROM) and pupillary reflexes.

The patient was managed with a comprehensive pharmacological regimen tailored to his condition. He received 100 mg of remdesivir daily through an IV line for antiviral therapy. For anticoagulation, 7500 units of unfractionated heparin was administered TDS in the subcutaneous route. Corticosteroid therapy included dexamethasone 8 mg IV BD. Antibiotic coverage was provided with co-amoxiclav 1 g IV TDS. These measures formed a multidisciplinary approach to address the complications of COVID-19 and manage comorbidities effectively.

During hospitalization, the patient's laboratory results indicated that his INR, PT, and PTT remained within the normal range (PTT: 26–30). Hemoglobin levels fluctuated, reaching a maximum of 11.8 and a minimum of 9.2. C-reactive protein (CRP) showed a decreasing trend, reflecting a reduction in inflammation, while lactate dehydrogenase (LDH) levels remained elevated above 1000 throughout his stay. D-dimer results were negative except on the final day, suggesting that thrombotic activity was generally low.

**Figure 3:**
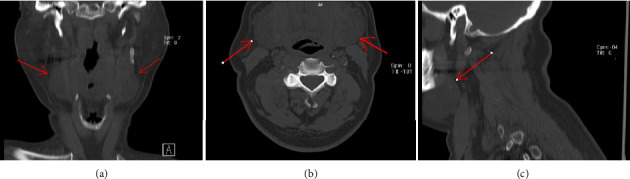
Nonenhanced CT scan of the face and neck shows bilateral heterogenous hypodense structures (red arrows) inferolateral to the tongue, extending to the midline. These structures measure approximately 48 × 37 × 27 mm on the left side and 43 × 40 × 22 mm on the right side, suggesting bilateral sublingual hematoma without significant airway compression at this stage. (a) Coronal view. (b) Axial view. (c) Parasagittal view.

**Figure 4:**
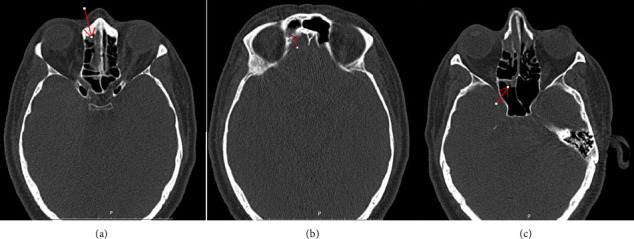
Partial opacification of (a) right frontal sinus (b), right ethmoidal air cells, and (c) right sphenoid sinus (red arrows).

Management focused on controlling blood pressure, as elevated blood pressure was suspected to contribute to bleeding. Cold compresses were applied, and the patient was kept in an upright, seated position. His anticoagulation therapy was held for 48 h as necessary to prevent further bleeding. A CT scan of the neck was performed to evaluate the hematoma (Figures 3 and 4).

**Figure 5:**
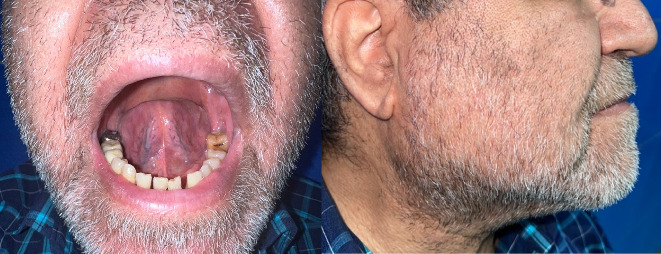
Follow-up photograph of the floor of the mouth after hematoma resolution. The floor of the mouth appears normal, with no visible swelling or discoloration, indicating successful conservative management and complete dissolution of the hematoma.

Given the risk of airway compromise, tracheostomy equipment was prepared and kept bedside. The anesthesia team was ready to perform a tracheostomy if surgical intervention became necessary, as intubation was considered unsafe in this scenario. The hematoma was closely monitored; its size initially increased but eventually stabilized and then gradually resolved without surgical intervention. The patient's respiratory symptoms and overall condition improved, allowing for his transfer out of the ICU (Figure 5).

**Figure 6:**
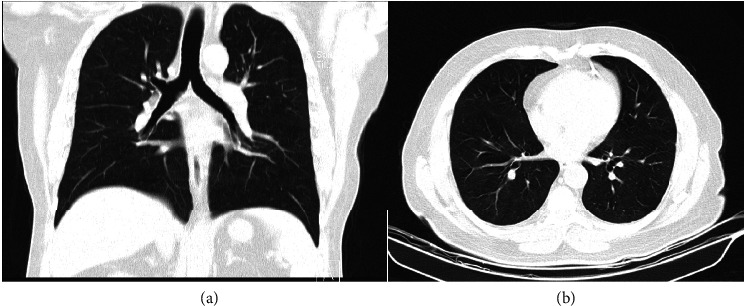
2 months after, in follow-up nonenhanced chest CT scan, alveolar opacities and pleural effusion are no longer visible.

The patient's condition remained stable following the resolution of the hematoma. He was subsequently discharged in good condition, with no further complications observed (Figure 6).

## 3. Discussion

Sublingual hematomas are rare but potentially life-threatening conditions that result from the rupture of blood vessels beneath the mucosa of the floor of the mouth, leading to swelling, pain, and sometimes airway obstruction. These hematomas can develop spontaneously, particularly in patients with underlying risk factors such as anticoagulant use, trauma, or systemic conditions that predispose to bleeding. Aggressive suctioning of the throat is another recognized risk factor for trauma and hematoma formation in the oral and pharyngeal regions. However, in this patient, no such interventions were performed, ruling it out as a contributing factor. Isolated sublingual hematoma is a rare and unexpected complication [9].

Sublingual varices are dilated veins located under the tongue, which can become prominent and susceptible to rupture due to increased venous pressure or weakened vessel walls. Factors leading to the development of sublingual varices include advanced age, cardiovascular diseases, and systemic conditions such as diabetes. Specifically, hypertension and high systolic blood pressure increase venous pressure, contributing to the formation of varices [10–12]. High fasting plasma glucose and low HDL levels, often seen in patients with diabetes, can weaken blood vessel walls [11]. In addition, abdominal obesity, male sex, older age, and smoking are significant risk factors [10–14]. The association between sublingual varices and these cardiovascular risk factors suggests that systemic pathological processes such as metabolic syndrome could influence their development [11–13, 15, 16]. Variceal hemorrhage occurs when these dilated veins rupture, leading to bleeding. This rupture can be triggered by elevated venous pressure, local trauma, or systemic conditions affecting vascular stability.

Hematomas in COVID-19 patients, particularly those on anticoagulant therapy, have been increasingly reported [17]. The combination of a hypercoagulable state and anticoagulation therapy creates a delicate balance between preventing thrombosis and avoiding hemorrhage. The use of heparin in COVID-19 patients is common due to the hypercoagulable state associated with the infection. Heparin helps prevent thromboembolic events, which are frequent in severe COVID-19 cases due to cytokine storms and SIRS [3].

A case report by Srikanth et al. further highlights the occurrence of sublingual hematoma as a rare but serious complication of anticoagulant therapy. In their report, a 75-year-old female patient developed a spontaneous sublingual hematoma after receiving heparin during coronary angioplasty, leading to significant airway obstruction. This complication, while rare, parallels the findings in our case, reinforcing the critical need for awareness among clinicians of the potential for such life-threatening events in patients receiving heparin, particularly when combined with other anticoagulants such as aspirin [18].

Adhikari et al. describe a similar case of a 55-year-old female on warfarin therapy who developed a spontaneous sublingual hematoma [9]. The clinical presentation included swelling below the tongue, similar to our case, and conservative management successfully resolved the condition. Likewise, Huang et al. reported a case of a 63-year-old man on apixaban who developed a sublingual hematoma following trauma, highlighting the rapid airway compromise associated with anticoagulant use and sublingual hematomas [19]. Furthermore, Buyuklu et al. described a 70-year-old woman who developed a spontaneous lingual and sublingual hematoma while on warfarin, with symptoms closely resembling those of our patient [20]. These cases emphasize the risk of spontaneous sublingual hematomas in anticoagulated patients, underscoring the need for prompt diagnosis and careful management to prevent airway compromise. Incorporating the use of laxatives in the management plan may help prevent recurrence of sublingual hematomas by minimizing straining during bowel movements, which can exacerbate venous pressure and increase the risk of rebleeding. This approach aligns with clinical guidelines that recommend stool softeners to reduce straining and associated complications in patients with bleeding disorders [21].

According to the anticoagulant test results, which remained within the normal range throughout the patient's treatment, two potential reasons can be suggested: First of all, based on concurrent evaluations conducted at the time on other patients undergoing anticoagulant therapy, regardless of their underlying conditions, the accuracy and reliability of the anticoagulant test results were unfortunately low. Even patients receiving therapeutic doses and continuous heparin drips showed test results that were within the normal range or slightly above it. This issue could be attributed to technical errors in sample collection, sample transportation, laboratory analysis, or the type of testing kits used. Second, the dosage of the anticoagulant medication prescribed to the patient may have been insufficient to induce significant changes in the laboratory test results.

In this case, several factors contributed to the patient's vulnerability to sublingual hematoma. His diabetes and sublingual varices increased the risk of vascular complications, while the use of heparin for COVID-19 added to the bleeding risk. Based on the existing scientific literature [22] and the fact that the patient was undergoing treatment with NPH insulin, the decision was made to refrain from using heparin's antidote due to the potential side effects and the possibility of exacerbating the patient's condition through increased sensitivity to protamine. In addition, his underlying cardiovascular issues, including significant coronary artery calcification and the presence of a coronary artery stent, further complicated his clinical picture. These factors necessitated meticulous balancing of anticoagulation therapy to prevent stent thrombosis while minimizing the risk of hemorrhage. This case highlights the need for a multidisciplinary approach to managing such complex patients, ensuring both thromboembolic prevention and bleeding risk mitigation.

## 4. Conclusion

In conclusion, spontaneous sublingual hematomas, though rare, present significant clinical challenges due to their potential to cause airway obstruction and complications in patients on anticoagulant therapy or with underlying systemic conditions. The cases reviewed highlight the diverse etiologies and management strategies for sublingual hematomas, emphasizing the importance of individualized patient care. Effective management often involves a multidisciplinary approach, balancing the risks of thrombosis and hemorrhage. Further research is warranted to establish standardized guidelines for the diagnosis and treatment of sublingual hematomas to improve patient outcomes and minimize complications.

## Data Availability

The data that support the findings of this study are available from the corresponding author upon reasonable request.
